# Whole Body Vibration Retards Progression of Atherosclerosis via Insulin-Like Growth Factor 1 in Apolipoprotein E-Deficient Mice

**DOI:** 10.1155/2018/4934861

**Published:** 2018-03-07

**Authors:** He Wu, Yibo Zhang, Xuan Yang, Xian Li, Zhenya Shao, Zipeng Zhou, Yuanlong Li, Shuwen Pan, Chang Liu

**Affiliations:** ^1^Department of Endocrinology, First Affiliated Hospital of Jinzhou Medical University, Jinzhou, Liaoning, China; ^2^Department of Pathogenic Biology, Jinzhou Medical University, Jinzhou, Liaoning, China; ^3^Schools of Nursing, Jinzhou Medical University, Jinzhou, Liaoning, China; ^4^Department of Orthopedics, Third Affiliated Hospital of Jinzhou Medical University, Jinzhou, Liaoning, China; ^5^Department of Orthopedics, First Affiliated Hospital of Jinzhou Medical University, Jinzhou, Liaoning, China

## Abstract

Whole body vibration (WBV) has a marked impact on lipid metabolism and the endocrine system, which is related to the progression of atherosclerosis (AS). To investigate the effects of WBV, we measured the atherosclerotic plaque area of apolipoprotein E-knockout (ApoE^−/−^) AS mice, which were trained by WBV (15 Hz, 30 min) for 12 weeks. Simultaneously, serum levels of lipids, insulin-like growth factor 1 (IGF-1), insulin-like growth factor 1 receptor (IGF-1R), interleukin 6 (IL-6), and the mRNA and protein levels of the same in the aorta were compared between the control and WBV groups. The results indicated that WBV significantly reduced the atherosclerotic plaque area with lower very low-density lipoprotein (VLDL) and oxidized low-density lipoprotein (ox-LDL) in the blood. Moreover, the levels of IGF-1 in serum and expression of IL-6, IGF-1R, and p-IGF-1R protein in the mice aorta decreased significantly in the WBV group. In addition, we found that serum IGF-1 in mice increased to the highest concentration in 30 min after WBV for 10, 30, 60, and 120 minutes. These results suggested that appropriate WBV may delay the progression of AS, which was associated with acutely elevated serum IGF-1 and lower levels of IGF-1 and IL-6 in the aorta for long-term treatment.

## 1. Introduction

Atherosclerosis (AS) is a long-term chronic disease characterized by deposition of subendothelial lipoproteins in the arterial wall of medium and large arteries [1]. The development of AS is a complex process including lipid infiltration, endometrial injury, inflammatory response, oxidative stress, and smooth muscle proliferation [2]. First, the accumulation of LDL-infiltrated lipid into the vascular endothelium through oxidative modification to form ox-LDL leads to arterial wall endothelial injury [3]. Thus, the deposition of subendothelial lipoproteins currently is recognized as the AS-initiating factor. Second, ox-LDL can stimulate vascular endothelial cells or induce macrophages to form foam cells, which produce abundant amount of proinflammatory cytokines [4, 5]. Subsequently, high levels of inflammatory factors such as TNF-*α* and IL-6 would induce the proliferation of smooth muscle cells and finally form atherosclerotic plaques. Consequently, reduction of high blood lipid and inflammation should be an effective strategy to delay the long-term process of atherosclerotic plaque formation.

Insulin-like growth factor 1 (IGF-1) is a circulating hormone in blood; binding of growth hormone (GH) to its hepatic receptor stimulates the expression and release of IGF-1 and plays an anti-inflammatory role in atherosclerotic plaque formation [6]. The role of IGF-1 is mediated by IGF-1R, which has an intrinsic tyrosine kinase activity that initiates downstream mediators including insulin receptor substrate 1 (IRS-1), phosphatidylinositol 3-kinases (PI3Ks), and mitogen-activated protein kinase (MAPK) [7]. Activation of the MAPK pathway causes phosphorylation of extracellular regulated protein kinase (ERK), promotes cell proliferation, and decreases apoptosis [8]. Thus, persistent exposure to IGF-1 may stimulate vascular smooth muscle cell (VSMC) overgrowth and migration, which is known to increase intimal thickness and restenosis [5]. In addition, recent studies have indicated that intense walking exercise decreased serum levels of IGF-1 and IGFBP3 as well as metabolic biomarkers including high-density cholesterol, glucose, and triglycerides [9].

Whole body vibration (WBV) is a novel type of physical training that increases muscle power. WBV requires less time and reaches higher compliance in inactive patients than traditional training regimens. Moreover, many traditional forms of physical activity may not be suitable for the elderly owing to debilitation, increase in risk of injury, or lack of motivation. In a previous study, we found that WBV might decrease lipid metabolism and inflammatory in fat rats [10] and improve metabolic issues by suppressing the reduction of IRS1, AKT2, and GLUT4 under diabetic conditions [11]. Vibration training can increase the anti-inflammatory factors—NO and TGF-*β*—in blood and promote the release of GH in humans [12, 13]. Meanwhile, sample data indicate that WBV can affect the release of growth hormones such as IGF-1 [14]. Therefore, we hypothesized that WBV could lessen the development of AS and improve exercise tolerance in some patients who lose their ability to move independently.

In this study, we established an AS model in ApoE^−/−^ mice and trained them with WBV (15 Hz, 30 min) for 12 weeks. The Oil red-O staining results showed that the AS plaque areas in the aorta were decreased in the WBV group. In addition, WBV exercise reduced the level of blood glucose, LDL, VLDL, ox-LDL, and IGF-1 in the serum of mice. Moreover, lower expression of IL-6 and IGF-1R was associated with the reduction of AS plaques in the WBV group.

## 2. Materials and Methods

### 2.1. Animals

ApoE^−/−^ mice were bred in the Laboratory Animal Center of Jinzhou Medical University, Liaoning, China (certificate number: SCXK2011-0015), according to institutional and government guidelines and approved by the local council of ethics. ApoE^−/−^ mice with the C57BL/6 genetic background were procured from Nanjing Laboratory Animals Center (SCX(SHU)2015-0001) and housed in a room with controlled temperature (22°C) and humidity (55–65%) under a light-dark cycle (lights were switched on at 9:00 am and switched off at 21:00 pm) in specific pathogen-free (SPF) conditions. Tap water and vacuum-packed pelleted food were provided ad libitum. Short-term vibration mice were randomly divided into control, 10 min, 30 min, 60 min, and 120 min groups (*n* = 8 animals/group), and blood samples were collected for further analysis after WBV (frequency: 15 Hz; acceleration: 0.68 g; amplitude: 2 mm).

Sixteen male mice (8 weeks old, 20–23 g) were randomly divided into 2 groups after being fed western diets (H10141, 0.15% cholesterol, provided by Beijing HFK Bioscience Co., Ltd., China) for 8 weeks in the abovementioned conditions, and the WBV group was trained for 30 min once a day in an LD-P vertical vibration machine (Huanzhen Machinery Limited Company, Guangdong, China; frequency: 15 Hz; acceleration: 0.68 g; amplitude: 2 mm). Vibration intervention was carried out from Monday to Saturday, with Sunday being a rest day. The training session began regularly at 9:00 am and lasted for 12 weeks. Then, mice from all groups were weighed after fasting for 12 h, blood samples were collected, and the serum was stored at −80°C. Aortas were collected and quickly stored in 4% paraformaldehyde or stored at −80°C until further analysis. Fasting blood glucose (FBG) in serum was measured by the glucose oxidase method once a month.

### 2.2. Methods

#### 2.2.1. Blood Biochemical Analysis

Blood samples were obtained from the angular vein system, and serum was separated by centrifugation at 3000 rpm for 15 min at 4°C and stored at −80°C until analysis. Triglycerides (TG), total cholesterol (TC), high-density lipoprotein cholesterol (HDL), low-density lipoprotein cholesterol (LDL), very low-density lipoprotein (VLDL), and oxidized low-density lipoprotein (ox-LDL) were measured by commercially available kits (Nanjing Jiancheng Bioengineering Institute, Nanjing, China) following manufacturers' instructions. Serum IGF-1 was assayed by ELISA (ab108874, Abcam, Cambridge, MA, USA). The kits were used according to the manufacturer's protocol.

#### 2.2.2. Quantitative Analysis of AS Lesions

After removing the adventitial fatty tissue, aortas were opened longitudinally from the aortic root to the iliac artery, fixed in 4% formalin for 36 h, and stained with Oil red O (O9755-25G Sigma-Aldrich, USA). To quantify the area of the AS lesion, the stained aortas were photographed using a digital camera connected to a dissection microscope (BX-51; Olympus, Tokyo, Japan) and then evaluated as the ratio of the positive area to the total aortic area by Image Pro Plus (version 6.0, Media Cybernetics, USA).

For cross-sectional quantification of plaque progression, 5 *μ*m serial paraffin sections from the indicated sites of the aorta were stained with hematoxylin and eosin. The maximum cross-sectional plaque areas from 200 sections in one aorta per animal were analyzed by Image-Pro Plus 6.0 (*n* = 4). Fibrous cap thickness was quantified by choosing the largest necrotic core in a section and measuring the thinnest part of the fibrous cap.

#### 2.2.3. Western Blot

Aortic tissues were kept on ice in 250 *μ*l RIPA (E1WP106, EnoGene, China) buffer per 20 mg and then centrifuged at 12,000 rpm for 20 min at 4°C to obtain the aortic protein. The total protein concentration was measured using a bicinchoninic acid (BCA) protein assay kit (P0012, Beyotime Institute of Biotechnology, China). The extracted proteins were separated via sodium dodecyl sulfate-polyacrylamide gel electrophoresis (SDS-PAGE) and transferred onto polyvinylidene difluoride (PVDF) membranes (Millipore, Billerica, MA). The membranes were blocked in 5% nonfat dry milk in Tris-buffered saline-Tween-20 (TBST) solution for 60 min at room temperature. Anti-phospho-IGF-1R (1 : 1000, Abcam, ab39675, UK), anti-IGF-1R (1 : 500, Abcam, ab39398, UK), ERK (1 : 1000, Abcam, ab54230, UK), p-ERK (1 : 1000, Abcam, ab65142, UK), and anti-IL-6 (1 : 1000, Abcam, ab9324, UK) were used as the primary antibodies, with glyceraldehyde-3-phosphate dehydrogenase (GAPDH) (HC301, TRANSGEN BIOTECH, Beijing, China) as the internal reference, overnight at 4°C. Then, the membrane was incubated with secondary antibodies conjugated with horseradish peroxidase (1 : 10000, Santa Cruz Biotechnology, USA) for 1 h at room temperature. Following the second incubation, each membrane was washed for 30 min. Finally, the membrane was soaked in ECL reagent and exposed to lumino imaging analyzer FAS-1100 (Toyobo Corp., Osaka, Japan). Densitometry analysis was performed using Image J software (Version 1.43, Broken Symmetry Software, Bethesda, MD), and expression of the related proteins was normalized to the densities of the respective GAPDH bands.

#### 2.2.4. Real-Time Quantitative PCR

We used quantitative PCR to quantify mRNA expression of IGF-1R and IL-6. Trizol reagent was used to extract total RNA from the aortic samples and reverse transcribed according to the manufacturer's instructions. Briefly, the cycling conditions were 42°C for 15 min, 70°C for 5 s, and 4°C forever. Real-time PCR was performed using Power Up™ SYBR® Green Master Mixture (TAKARA, Japan) and detected with the LightCycler 480 (Roche, Switzerland) as follows: one cycle at 95°C for 30 min; 40 cycles at 95°C for 5 s, and 60°C for 30 s; and one cycle at 95°C for 15 s, 60°C for 30 min, and 95°C for 15 s. The primer sequences are listed in Table 1. The relative quantification values for these gene expressions were calculated by 2^−ΔΔCt^ method and corrected using a housekeeping gene—GAPDH—as reference.

### 2.3. Statistical Analysis

Graph Pad Software v5.0 (Graph Pad Software Inc., USA) was used for analyzing data, which were presented as means ± SEM. Statistical significance was evaluated using unpaired Student's *t*-test for comparisons between two groups. Groups were compared using one-way ANOVA and Tukey's post hoc tests. Differences with a value of *p* < 0.05 were considered statistically significant.

## 3. Results

### 3.1. WBV Affected Blood Glucose and Lipids in AS Mice

**Figure 1:**
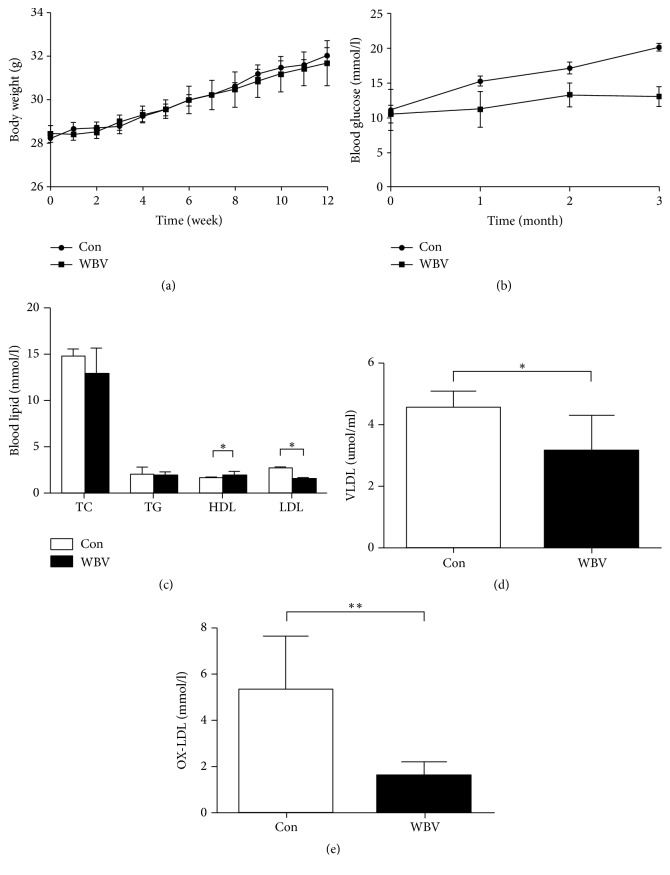
Body weight and lipid metabolism index in AS model mice treated with WBV. After 12 weeks of WBV training with 15 Hz, body weight at every week (a), blood glucose (b), blood lipids such as TC, TG, HDL, LDL (c), VLDL (d), and OX-LDL (e) were compared between the control and WBV groups. Data are expressed as means ± SEM (*n* = 8); ^*∗*^*p* < 0.05 versus control group; ^*∗∗*^*p* < 0.01 versus control group.

Previous studies have shown that exercise training such as swimming [15] and running wheel for mice [16] reduced AS lesion formation in mice models. In order to investigate the effect of WBV on the aortic plaque in mice, AS mice were trained by WBV for 12 weeks. In this study, there were no significant differences in the body weight between the control and WBV groups (*p* > 0.05; Figure 1(a)).  Figure 1(b) shows a significant reduction in blood glucose levels after WBV treatment was carried out for 1 month. Regarding blood lipids, WBV had no effect on TC, TG, and HDL concentrations in mice (Figure 1(c), *p* > 0.05); however, the level of LDL, VLDL, and ox-LDL, which are closely related to AS, significantly decreased in the WBV group (*p* < 0.05; Figures 1(c), 1(d), and 1(e)). The reduction of blood glucose and lipoproteins indicated that WBV had an effect on AS in the mice after 12 weeks.

### 3.2. WBV Decreased the Aortic Plaque Area in AS Mice

**Figure 2:**
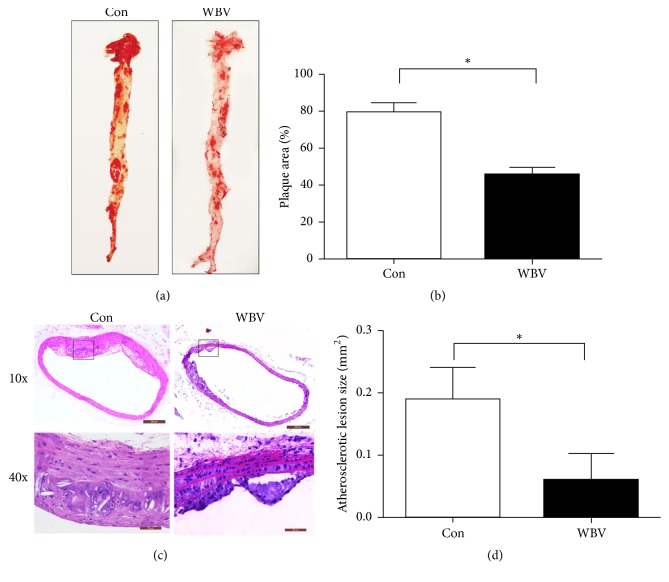
AS lesion formation in the aorta of ApoE^−/−^ mice. WBV reduced AS lesion formation in ApoE^−/−^ mice. (a) Aortic en face flat from the aortic root to the iliac artery stained with Oil red O. (b) The plaque size was measured as a percentage of the total aortic surface. Plaque-covered area was determined and expressed as a percentage of the total area. (c) Hematoxylin-eosin stains for morphometry. (d) Cross-sectional size of the aorta was quantified by manual outlining in the Image Pro software. Data are shown from representative experiments (*n* = 4). ^*∗*^*p* < 0.05 versus control group.

To determine whether atherosclerotic plaque formation was inhibited by chronic WBV training, we measured plaques on the luminal surface of whole aortas by Oil red-O staining and cross-sectional area of plaques in the thoracic aorta by HE staining. Oil red-O staining showed that there were several large atherosclerotic plaques throughout the aortic tree in the control group, particularly entire confluent plaques in the aortic arch area. In contrast, flat-mounted aortas of the WBV group exhibited incompletely fused plaques in the aortic arch and scattered small or thin plaques in the thoracoabdominal aorta (Figure 2(a)). Moreover, the percentage of plaques in the entire aorta in the WBV group was smaller (45.7%) than that of control group (79.6%) (Figure 2(b)).

Simultaneously, we measured the cross-sectional area of the aortic plaque to compare the degree of AS regression between the control and WBV groups. Morphological photographs showed that there was thicker plaque lesion in the control than the WBV group (Figure 2(c)). Besides, quantification of the AS lesion area showed that a 12-week WBV training significantly decreased the progression of AS lesions (Figure 2(d)). Consequently, long-term WBV training may delay the development of AS in ApoE^−/−^ mice.

### 3.3. WBV Reduced Inflammatory Factor IL-6 in the Aorta

**Figure 3:**
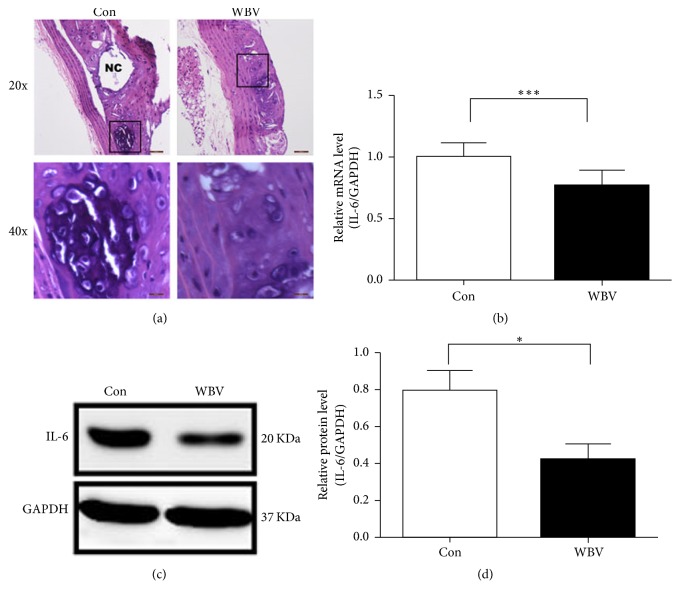
Histological analysis of plaque progression in the thoracic aorta after WBV for 12 weeks. (a) Morphological photographs and the foam cells of aortic lateral section after hematoxylin-eosin (HE) staining in control and WBV groups. The black box indicates foam cells and NC indicates necrotic core. (b) Quantitative real-time PCR (Q-PCR) analysis of mRNA levels of IL-6 in aorta. (c) Western blot analysis of IL-6 in mice aorta. IL-6 protein content is normalized to GAPDH protein content. (d) Quantification of western blot results of IL-6 levels expression in aorta. Values are means ± SEM; *n* = 4; ^*∗*^*p* < 0.05 versus control group; ^*∗∗∗*^*p* < 0.001 versus control group.

The persistence of foam cells (activated and lipid-laden macrophages) in the arterial wall fuels the development of AS. In this study, artery HE staining of the arterial cross-section showed that the aortic wall in the control group exhibited typical plaques with a necrotic core, which was infiltrated by numerous foam cells (Figure 3(a)). On the contrary, in the WBV group, the hyperplasia of the intima was mild with few fractures and only a small amount of lipid plaque and foam cells were visualized. This indicated that long-term intervention with WBV impeded foam cell formation in the aorta.

IL-6, which is released by foam cells, plays an important role in the progression of AS. Therefore, we detected the mRNA and protein expression of IL-6 in the aortas between the two groups. As is shown in Figures 3(b), 3(c), and 3(d), the mRNA and protein expression of IL-6 significantly decreased after WBV treatment. The above results showed that inflammatory reaction was reduced in the aortas with long-term WBV.

### 3.4. IGF-1/ERK in the Aorta Was Lowered by 12 Weeks of WBV

**Figure 4:**
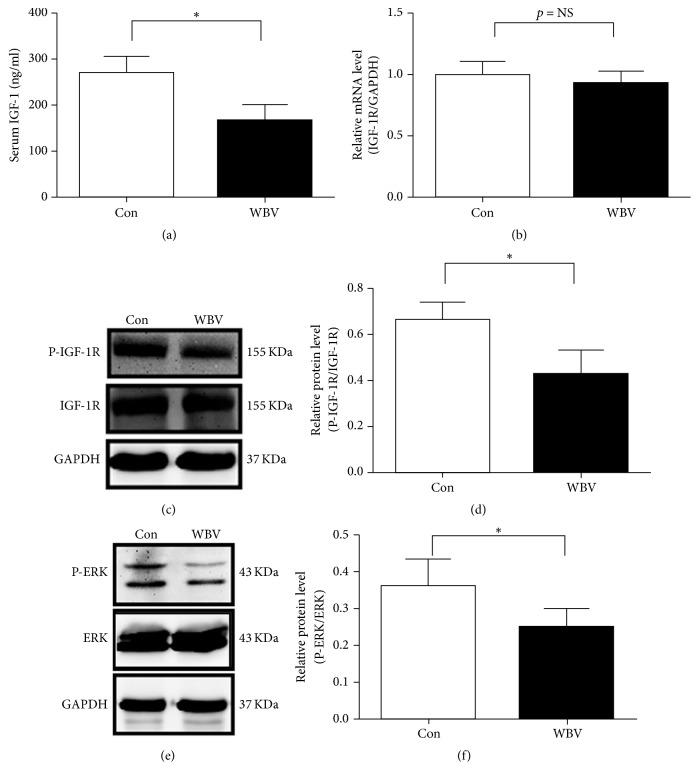
Effect of WBV for 12 weeks on the IGF-1-ERK sisgnaling pathway. (a) Serum IGF-1 levels were detected by ELISA after 12 weeks of vibration. (b) Q-PCR analysis of mRNA levels of IGF-1R in mice aorta. (c) Western blots analysis of IGF-1R and p-IGF-1R in mice aorta. (d) Quantification of the western blot analysis of p-IGF-1R expression in the aorta. (e) Protein expression of ERK and p-ERK in aortas after vibration for 12 weeks. (f) Western blot analysis of ERK and p-ERK proteins in ApoE^−/−^ mice. The expression of these proteins was quantified as relative expression after normalization to GAPDH expression. Values are means ± SEM, *n* = 4, and ^*∗*^*p* < 0.05 versus control group.

Experimental evidence indicates that IGF-1 reduces atherosclerotic plaque burden as an anti-inflammatory molecule [17]. Generally, there is an inverse relation between serum IL-6 and IGF-1 levels, and IGF-1/IGFBP-3 administration to patients with severe burn injury induced an anti-inflammatory effect and reduced IL-6 [18, 19]. We detected the level of IGF-1 in serum; it showed that serum IGF-1 decreased by 41.3% in the WBV group compared to the control group (*p* < 0.05; Figure 4(a)). To confirm whether decreased serum IGF-1 minimized its function in the aorta, we continued to assess the mRNA expression and phosphorylation of IGF-1R in the aorta and phosphorylation of ERK. RT-PCR results showed that IGF-1R expression was not significant; however, phosphorylation of IGF-1R in the WBV group was significantly lower than that in the control group (Figures 4(b)-4(d)). In addition, western blotting indicated that phosphorylation of ERK declined significantly in the WBV group. Taken together, long-term WBV reduced serum IGF-1, phosphorylation of IGF-1R in aortas, and ERK phosphorylated levels, which inhibit endothelial cell proliferation in blood vessels.

### 3.5. The Level of Plasma IGF-1 Increased after Short-Term WBV

**Figure 5:**
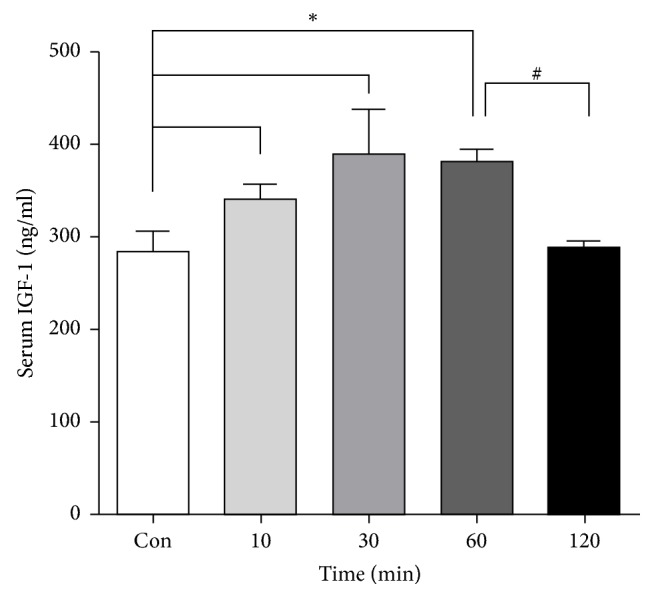
The level of serum IGF-1 at different times of WBV treatment. Serum IGF-1 levels were detected by ELISA after WBV for 10, 30, 60, and 120 min. Values are means ± SEM; *n* = 8, ^*∗*^*p* < 0.05 versus control group, and ^#^*p* < 0.05 versus 120 min group.

A recent study showed that WBV produced an acute increase in the circulating levels of IGF-1 in older individuals [14]. To assess the acute effects of WBV on serum IGF-1, we performed WBV on ApoE^−/−^ mice for 10, 30, 60, and 120 min, respectively, and then measured the IGF-1 concentration in the blood. We found that IGF-1 showed a tendency to rise first and then decline compared with the control group (Figure 5). Serum IGF-1 was increased by 27% after a 30-minute vibration exercise. However, after 2 h of vibration, serum levels decreased to as much as the control level. This indicated that WBV could increase serum IGF-1 if performed at the appropriate time.

## 4. Discussion

In the present study, we evaluated whether WBV could attenuate the development of AS in ApoE^−/−^ mice. Mice fed a western diet for 8 weeks showed AS lesions characterized by accumulation of SMCs and macrophages. The 12-week vibration training significantly reduced aortic plaque areas, serum LDL, VLDL, ox-LDL, and IGF-1 levels in the AS mice model. Moreover, IL-6, phosphorylation of IGF-1R, and ERK significantly declined in the aorta as well in the WBV group. However, acute induced WBV increased IGF-1 in the blood. These findings indicated that long-term WBV retarded AS progression, accompanied with lower level of lipoprotein and IGF-1 in the blood and lower level of inflammatory IL-6, and decreased IGF-1/ERK signaling in the aorta, whereas, short-term WBV increased IGF-1 in the blood.

AS is a chronic inflammatory disease in which oxidative stress plays a critical role not only in initial lesion formation but also in lesion progression and destabilization. The accumulation of lipids in endothelial cells and macrophages in the aorta leads to oxidative stress, which is associated with abnormalities in blood glucose, blood lipids, and blood lipoproteins. In our study, the vibration movement did not significantly reduce TC and TG in the AS mice, although the previous study showed that WBV significantly reduced elevated levels of TC and TG in obese mice [20]. However, as known, the level of blood lipoproteins is more relative to AS initiation. For instance, ox-LDL can promote AS foam cell formation [21], damage vascular endothelial cells [22], stimulate vascular smooth muscle cell proliferation [23], and enhance inflammatory cytokine release [24]. In addition, Bravo Monteiro et al. showed that rats exposed to short-term WBV of 20 Hz had reduced plasma VLDL levels [25]. Thus, we measured the blood lipoproteins and found that WBV significantly decreased the level of LDL, VLDL, and ox-LDL in ApoE^−/−^ mice. Numerous studies have confirmed that prolonged or high-intensity exercise results in oxidative damage to macromolecules in both blood and skeletal muscle [26]; however, moderate intensity exercise training reduced the levels of ox-LDL [27]. Therefore, the reduction of lipoproteins, particularly ox-LDL, might be important indices for the application of WBV in individuals.

IL-6 is a pleiotropic proinflammatory cytokine and has been reported to serve as a valuable indicator of atherogenesis and instability of AS plaques [28]. IL-6 is produced by a variety of cell types such as T-cells and macrophages, but IL-6 is also readily produced by other cell types such as SMCs. In this study, we found that there were a large number of foam cells and high level of inflammatory IL-6 in AS plaques, reduced by long-term WBV. In muscle cells, IL-6-induced proliferation and migration occur via the JAK/STAT pathway, which stimulates the expression of proliferation-associated transcription factors cyclin D1 and c-myc [29]. Accordingly, a decrease in the spontaneous release of IL-6 induced by physical exercise [30] or WBV would exert an influence on VSMC overgrowth and migration. The decreased IL-6 is beneficial to slow the progression of AS after WBV.

IGF-1 is also a potent survival factor for VSMC, which is synthesized by almost all tissues. Transcription factors have been identified to demonstrate an overlap between IL-6 and IGF in the cell replication cycle and include the Janus kinase/signal transducer activator of transcription (JAK/STAT) pathway and suppressors of cytokine signaling (SOCS) [31]. For example, infusion of IL-6 has been shown to increase IGF-1 sensitivity in acute scenarios [32]. To our knowledge, increased IGF-1 signaling prevents ox-LDL-induced VSMC apoptosis [33]. Thus, high levels of serum IGF-1 are considered beneficial in preventing the development of AS. Contrary to the expectation, however, long-term WBV treatment (12 weeks) markedly decreased serum IGF-1 levels in our study. IGF-1 exerts its physiologic effects by binding to the IGF-1 receptor (IGF-1R), which initiates the IRS-1, PI3K, and MAPK pathway and activates ERK to promote cell proliferation and survival [34]. To verify the IGF-1 function, we evaluated the mRNA level and phosphorylation of IGF-1 and ERK in aortas. The results showed that the higher phosphorylation of IGF-1R and ERK in the control group due to severe atherosclerotic lesions retrieved ox-LDL–induced injury in the aorta but promoted cell proliferation. Hence, low levels of IGF-1 could be beneficial in retarding the progression of AS in early stages.

On the contrary, other studies indicated that increased circulating IGF-1 reduces systemic and vascular oxidant stress, vascular cytokine expression, and AS progression [35]. For vibration training, Cardinale et al. found that short-term 5-minute vibration training at 30 Hz significantly increased the levels of IGF-1 in the elderly but had no effect on the young population [14]. Moreover, we also found that the serum IGF-1 concentration firstly increased reaching the highest concentration at 30 min and then decreased with the extension of vibration time. The acute effect of WBV on IGF-1 suggested that WBV increased serum IGF-1 at the appropriate time. Our study showed that WBV induced level change of IGF-1 was linked to AS progression, but the antiatherosclerotic exact mechanisms IGF-1 requires further clarification. Moreover, appropriate serum IGF-1 should be defined when an individual with AS was treated with WBV.

## 5. Conclusions

Our study confirms that WBV can attenuate the development of AS and lead to lower levels of LDL, VLDL, and ox-LDL in the blood, as well as IL-6 expression in the aorta. In addition, IGF-1 level plays an important role in WBV-treated AS model and may be used to monitor the effect of WBV in patients.

## Figures and Tables

**Table 1:** Primer sequences for real-time PCR.

Gene	Upstream primer (5′-3′)	Downstream primer (5′-3′)	Amplified size of the fragment (bp)
GAPDH	TGGTGAAGGTCGGTGTGAAC	GCTCCTGGAAGATGGTGATGG	231
IGF-1R	GTGAACGCCCATCTGGGAAG	CCAGGGGCAGCCATTAAGTC	74
IL-6	CTGCAAGAGACTTCCATCCG	AGTGGTATAGACAGGTCTGTTGG	131

GAPDH: glyceraldehyde-3-phosphate dehydrogenase; IGF-1R: insulin-like growth factor 1 receptor; IL-6: interleukin 6.
